# Supplementary material for: Dietary Patterns and the Double Burden of Malnutrition in Mexican Adolescents: Results from ENSANUT-2006
Source: Nutrients. 2019 Nov 13;11(11):2753. doi: 10.3390/nu11112753 (PMC6893405; doi:10.3390/nu11112753)
Supplement: Supplementary file 1 [file nutrients-11-02753-s001.pdf]

Supplementary Table S1: Food groups, examples of food items and percentage of consumers per food group.

| Food group | Examples | % consumers |
|---|---|---|
| 1. Tortilla | Maize tortilla | 90.2 |
| 2. Cereals (except corn) | Potatoes, bread, wheat tortilla, salty cookie | 63.6 |
| 3. Breakfast cereals | Breakfast cereals with low and high fiber content | 30.1 |
| 4. Maize based food | Tamales, pozole, taco, tlacoyo, sope, atole (maize-drink) | 49.1 |
| 5. Fast food | Hotdog, pizza, hamburger | 13.1 |
| 6. Alcoholic drinks | Beer, wine, spirits | 4.2 |
| 7. Legumes | Beans, lentils, chick peas, soy | 90.0 |
| 8. Fruit | Apple, banana, grapes, grapefruit, kiwi, lemon, melon, papaya, pear, pineapple, strawberry, watermelon, others. | 93.3 |
| 9. Vegetables | Bell pepper, carrots, chili pepper, col, corn, cucumber, dark green vegetables, jicama, lettuce, tomato, zucchini, others. | 97.6 |
| 10. Poultry and red meat | Chicken, beef, pork, lamb, entrails | 86.4 |
| 11. Fish and sea food | Tuna, sardines, dry fish, fresh fish, sea food | 34.4 |
| 12. Processed meat | Ham, sausages, bacon, chorizo | 45.5 |
| 13. Milk | Fat free and low fat milk, whole milk | 68.5 |
| 14. Eggs | | 75.8 |
| 15. Saturated fat | Oil, butter, margarine, mayonnaise, cream | 34.2 |
| 16. Sugar and sweets | Sugar, candies, chocolate, jelly | 74.3 |
| 17. Cookies | Sweet cookies, granola bars | 32.6 |
| 18. Salty snacks | Corn snacks and chips | 51.0 |
| 19. Industrialized sweet-drinks | All types of soda and industrialized juice | 84.9 |
| 20. Non-industrialized sweet-drinks | Natural juice, water with sugar, coffee or tea with sugar | 83.3 |
| 21. Milk with added sugar | Milk with sugar, honey or chocolate, Yakult | 5.9 |
| 22. Drinks without energy | Coffee or tea without sugar, water, diet sodas | 8.8 |
| 23. Dairy products | Yogurt, cheese | 70.5 |
| 24. Sandwich | Sandwich and torta (baguette type) | 36.5 |
| 25. Fried dishes (plant based) | Breaded or capped vegetables, fried banana, fried potatoes, potato-tortita | 28.5 |
| 26. Sweet-bakery | Cakes, sweet bread, doughnut, churros, muffin | 60.5 |
| 27. Supplements | NUTRISANO and NUTRIVIDA (dietary supplements administrated by the health ministry) | 0.3 |
| 28. Soups | Vegetable soup, vegetables cream, fideo soup | 77.3 |
| 29. Dry soups | Rice, pasta | 68.6 |
| 30. Avocado and nuts | Avocado and oily seeds (nuts, almond, soy) | 39.0 |

Supplementary Table S2: Prevalence ratios of overweight-obesity and stunting, and dietary patterns among Mexican adolescents. Not adjusted for EI:BMR ratio

| Dietary pattern | Total * | 12-15 years § (n=4,451) | 16-19 years § (n=2,929) | Boys ¥ (n=3,610) | Girls ¥ (n=3,770) |
|---|---|---|---|---|---|
| | | | Overweight and obesity | | |
| Non-traditional & breakfast/lunch-type | 1.05 (0.99-1.11) | 1.12 (1.03-1.21) | 0.93 (0.84-1.02) | 1.02 (0.94-1.11) | 1.07 (0.99-1.16) |
| Western | 1.05 (0.99-1.10) | 1.09 (1.01-1.17) | 0.96 (0.88-1.06) | 1.13 (1.04-1.24) | 0.98 (0.90-1.06) |
| Plant-based | 0.95 (0.90-1.00) | 0.96 (0.89-1.04) | 0.93 (0.85-1.02) | 0.96 (0.88-1.04) | 0.95 (0.88-1.02) |
| Protein-rich | 0.94 (0.89-0.99) | 0.92 (0.86-0.98) | 0.98 (0.88-1.08) | 0.99 (0.90-1.09) | 0.90 (0.83-0.97) |
| | | | Stunting | | |
| Non-traditional & breakfast/lunch-type | 0.86 (0.79-0.94) | 0.87 (0.78-0.98) | 0.85 (0.76-0.94) | 0.87 (0.80-0.95) | 0.86 (0.75-0.97) |
| Western | 0.91 (0.84-0.98) | 0.94 (0.85-1.04) | 0.89 (0.79-0.99) | 0.90 (0.81-1.01) | 1.01 (0.91-1.11) |
| Plant-based | 0.95 (0.90-1.00) | 0.96 (0.89-1.04) | 0.93 (0.85-1.02) | 0.96 (0.88-1.04) | 0.90 (0.83-0.98) |
| Protein-rich | 1.01 (0.95-1.07) | 0.98 (0.90-1.08) | 1.03 (0.95-1.12) | 1.05 (0.95-1.16) | 1.05 (0.95-1.17) |

* Model adjusted for sex, living area, socioeconomic status, region, age § Model adjusted for sex, living area, socioeconomic status, region and ¥ Model adjusted for living area, socioeconomic status, region, age. None of the Prevalence Ratios were adjusted for EI:BMR ratio, for adjusted prevalence ratios see Table 4 and 6.

Supplementary Table S3: Total energy and nutrient intake across quartiles (Q) of the four dietary patterns.

| | Quartile of dietary pattern score | | | | |
|---|---|---|---|---|---|
| | $Q_1$ | $Q_2$ | $Q_3$ | $Q_4$ | *p-value* |
| Non-traditional & breakfast/lunch-type | | | | | |
| EI:BMR ratio (SD) | 1.08 (0.42) [2,3,4] | 0.85 (0.37) [1,4] | 0.88 (0.37) [1,4] | 1.01 (0.40)[1,2,3] | *<0.0001* |
| Energy kcal (SD) | 2035.1 (805.4)[2,3,4] | 1602.8 (688.0) [1,4] | 1627.6 (711.1) [1,4] | 1935.5 (774.6)[1,2,3] | *<0.0001* |
| Protein % (SD) | 10.4 (1.7) [2,3,4] | 10.7 (2.1) [1,3,4] | 11.6 (2.2) [1,2,4] | 12.6 (2.4) [1,2,3] | *<0.0001* |
| Fat % (SD) | 22.3 (8.2)[2,4] | 24.9 (7.7)[1,3,4] | 27.7 (7.1)[2,4] | 29.6 (6.3)[1;2;3] | *<0.0001* |
| Carbohydrates % (SD) | 67.2 (8.8)[2;3;4] | 64.1 (8.6) [1,4] | 60.6 (8.2) [1,4] | 57.7 (7.7) [1;2;3] | *<0.0001* |
| Fiber g (SD) | 27.3 (12.3)[2,3,4] | 19.4 (9.1)[1,3,4] | 17.4 (9.4) [1,2] | 18.4 (9.5) [1,2] | *<0.0001* |
| Sugar g (SD) | 13.7 (20.0)[3,4] | 13.5 (20.2)[3,4] | 16.5 (22.8)[1,2,4] | 21.2 (23.9)[1,2,3] | *<0.0001* |
| Ca mg (SD) | 885.2 (450.4)[2,3,4] | 677.9 (364.6)[1,3,4] | 731.3 (388.0)[1,2,4] | 1075.7 (472.4)[1,2,3] | *<0.0001* |
| Fe mg (SD) | 13.7 (5.8)[2,3,4] | 9.9 (4.9)[1,4] | 9.9 (5.2)[1,4] | 12.6 (6.2)[1,2,3] | *<0.0001* |
| Zn mg (SD) | 7.6 (3.3)[2,3,4] | 6.0 (3.2)[1,3,4] | 6.5 (3.1)[1,2,4] | 8.6 (3.8)[1,2,3] | *<0.0001* |
| Vit C mg (SD) | 54.7 (78.0) [2,3,4] | 60.7 (78.1) [1,3,4] | 70.2 (80.1) [1,2,4] | 97.0 (93.4) )[1,2,3] | *<0.0001* |
| Vit A | 2306.0 (3262.2)[2,3,4] | 2642.2 (3414.9) [1,3,4] | 3162.8 (3542.6) [1,2,4] | 4928.1 (4612.2) [1,2,3] | *<0.0001* |
| Folate | 236.1(132.2)[2,3,4] | 192.7 (109.0)[1,4] | 197.9 (111.2)[1,3] | 249.6(126.5)[1,2,3] | *<0.0001* |
| Vit B12 | 1.3 (1.4) [2,3,4] | 1.5 (1.5) [1,3,4] | 2.0 (1.5) [1,2,4] | 3.2 (2.1) )[1,2,3] | *<0.0001* |
| Western | | | | | |
| EI:BMR ratio (SD) | 0.85 (0.37)[3,4] | 0.85 (0.35)[3,4] | 0.95 (0.37)[1,2,4] | 1.189 (0.42)[1,2,3] | *<0.0001* |
| Energy kcal (SD) | 1558.5 (677.8)[3,4] | 1562.8 (647.4) [3,4] | 1796.4 (689.5)[1,2,4] | 2301.3 (807.4)[1,2,3] | *<0.0001* |
| Protein % (SD) | 11.2 (2.0) [,4] | 11.3 (2.2) [,4] | 11.3 (2.4) [,4] | 11.1 (2.4) [1,2,3] | *<0.0001* |
| Fat % (SD) | 21.3 (7.4)[2,3,4] | 25.0 (7.0) [1,3,4] | 28.1 (7.4)[1,2,4] | 31.1 (6.6) [1,2,3] | *<0.0001* |
| Carbohydrates % (SD) | 67.2 (8.6) [2,3,4] | 63.6 (8.3) [1,3,4] | 60.5 (8.4) [1,2,4] | 57.6 (8.2) [1,2,3] | *<0.0001* |
| Fiber g (SD) | 21.7 (10.97)[2,3,4] | 19.4 (10.3)[1,4] | 19.7 (11.0) [1,4] | 20.6 (11.2) [1,2,3] | *<0.0001* |
| Sugar g (SD) | 8.2 (13.2) [2,3,4] | 13.7 (17.1) [1,3,4] | 19.2 (21.0) [1,2,4] | 29.3 (27.5) [1,2,3] | *<0.0001* |
| Ca mg (SD) | 837.2 (444.1)[2,4] | 762.1 (407.8)[1,3,4] | 808.4 (448.1)[2'4] | 896.8 (474.8)1,2,3 | *<0.0001* |
| Fe mg (SD) | 10.2 (5.4)[3,4] | 9.9 (5.1) [3,4] | 11.4 (5.4)[1,2,4] | 14.3 (6.1)[1,2,3] | *<0.0001* |
| Zn mg (SD) | 6.2 (3.0) [3,4] | 6.2 (3.0) [3,4] | 7.2 (3.3) [1,2,4] | 9.2 (3.8) [1,2,3] | *<0.0001* |
| Vit C mg(SD) | 77.8 (96.2)[2,3] | 61.8 (80.2)[1,4] | 66.3 (74.5) [1,4] | 77.5 (82.3) [2,3] | *<0.0001* |
| Vit A | 3600.3 (4593.7) [2,3] | 2961.0 (3604.7) [1,4] | 2922.1 (3594.3) [1,4] | 3351.3 (3642.3) [2,3] | *<0.0001* |
| Folate | 201.9 (118.8) [3,4] | 197.0 (111.4) [3,4] | 218.6 (118.5) [1,2,4] | 257.0 (132.9) [1,2,3] | *<0.0001* |
| Vit B12 | 1.3 (1.5)[2,3,4] | 1.6 (1.5)[1,3,4] | 2.0 (1.7)[1,2,4] | 3.0 (2.0)[1,2,3] | *<0.0001* |
| Plant-based | | | | | |
| EI:BMR ratio (SD) | 0.75 (0.30)[2,3,4] | 0.85 (0.33)[1,3,4] | 0.99 (0.35)[1,2,4] | 1.31 (0.41)[1,2,3] | *<0.0001* |
| Energy kcal (SD) | 1423.2 (589.0) [2,3,4] | 1581.0 (608.9)[1,3,4] | 1846.3 (656.1)[1,2,4] | 2448.2 (796.0)[1,2,3] | *<0.0001* |
| Protein % (SD) | 11.6 (2.5) [2,3,4] | 11.3 (2.2) [1,3,4] | 11.1 (2.2) [1,2,4] | 10.9 (2.2 [1,2,3] | *<0.0001* |
| Fat % (SD) | 27.4 (8.4) [2,3,4] | 26.0 (7.8) [1,3,4] | 26.2 (7.7) [1,2,4] | 26.5 (7.1) [1,2,3] | *<0.0001* |
| Carbohydrates % (SD) | 60.9(9.9) [2,3,4] | 62.5 (8.9 )[1,3,4] | 62.1 (8.8) [1,2,4] | 62.5 (8.2) [1,2,3] | *<0.0001* |
| Fiber g (SD) | 15.8 (8.6) [2,3,4] | 18.1 (9.2)[1,3,4] | 21.0 (9.7) [1,2,4] | 28.1 (11.7) [1,2,3] | *<0.0001* |
| Sugar g (SD) | 9.2 (19.5) [2,3,4] | 13.7 (20.5) [1,3,4] | 17.2 (21.3) [1,2,4] | 24.6 (24.2) [1,2,3] | *<0.0001* |
| Ca mg (SD) | 643.5 (356.7) [2,3,4] | 744.2 (382.2)[1,3,4] | 852.8 (411.0) [1,2,4] | 1122.3 (485.2) [1,2,3] | *<0.0001* |
| Fe mg (SD) | 9.5 (4.8) [2,3,4] | 10.0 (5.0)[1,3,4] | 11.6 (5.2) [1,2,4] | 15.2 (6.2) [1,2,3] | *<0.0001* |
| Zn mg (SD) | 5.9 (2.8) [2,3,4] | 6.4 (2.9) [1,3,4] | 7.2 (3.2) [1,2,4] | 9.6 (3.8) [1,2,3] | *<0.0001* |
| Vit C mg (SD) | 35.9 (45.9) [2,3,4] | 54.5 (51.2) [1,3,4] | 79.8 (69.4) [1,2,41,2,4] | 147.3 (104.2) [1,2,3] | *<0.0001* |
| Vit A | 1719.7 (1874.3) [2,3,4] | 2500.5 (2606.6) [1,3,4] | 3667.1 (3283.1) [1,2,4] | 6309.7 (4908.1) [1,2,3] | *<0.0001* |
| Folate | 178.3 (107.8) [2,3,4] | 191.0 (102.8) [1,3,4] | 218.3 (110.9) [1,2,4] | 296.3 (130.7) [1,2,3] | *<0.0001* |
| Vit B12) | 1.6 (1.5) [3,4] | 1.7 (1.5) [3,4] | 2.0 (1.8) [1,2,4] | 1.9 (1.8) [1,2,3] | *<0.0001* |
| Protein-rich | | | | | |
| EI:BMR ratio | 0.83 (0.36)[2,3,4] | 0.88 (0.37)[1,3,4] | 0.97 (0.39)[1,2,4] | 1.17 (0.42)[1,2,3] | *<0.0001* |
| Energy kcal (SD) | 1434.2 (662.0)[2,3,4] | 1637.2 (688.2)[1,3,4] | 1805.05 (736.4)[1,2,4] | 2226.1 (803.3)[1,2,3] | *<0.0001* |
| Protein % (SD) | 10.3 (2.2) [2,3,4] | 11.0 (2.2) [1,3,4] | 11.5 (2.1) [1,2,4] | 12.0 (2.2) [1,2,3] | *<0.0001* |
| Fat % (SD) | 25.3 (8.2) [,3,4] | 25.4 (7.7) [,3,4] | 27.0 (7.5) [1,2,4] | 28.1 (7.3) [1,2,3] | *<0.0001* |
| Carbohydrates % (SD) | 64.1 (9.2) [2,3,4] | 63.5 (8.8) [1,3,4] | 61.4 (8.6) [1,2,4] | 59.7 (8.6) [1,2,3] | *<0.0001* |
| Fiber g (SD) | 15.9 (8.7) [2,3,4] | 18.5 (9.1) [1,3,4] | 21.2 (10.3) [1,2,4] | 27.0 (11.0) [1,2,3] | *<0.0001* |
| Sugar g (SD) | 18.1 (23.1)[2,3] | 14.2 (20.5)[4,1] | 15.5 (22.1)[4,1] | 17.2 (22.3) [2,3] | *<0.0001* |
| Ca mg (SD) | 726.1 (402.2) [2,3,4] | 769.3 (427.9) [1,3,4] | 834.1 (440.7) [1,2,4] | 973.5 (476.5) [1,2,3] | *<0.0001* |
| Fe mg (SD) | 9.3 (5.0) [2,3,4] | 10.3 (5.0) [1,3,4] | 11.9 (5.3) [1,2,4] | 14.8 (6.2) [1,2,3] | *<0.0001* |
| Zn mg (SD) | 5.7 (2.9) [2,3,4] | 6.5 (2.9) [1,3,4] | 7.4 (3.3) [1,2,4] | 9.5 (3.8) [1,2,3] | *<0.0001* |
| Vit C mg (SD) | 56.1 (73.9) [2,3,4] | 64.4 (78.5) [1,3,4] | 76.7 (83.8) [1,2,4] | 85.6 (95.0) [1,2,3] | *<0.0001* |
| Vit A | 2257.0 (3323.2) [2,3,4] | 2773.0 (3267.6) [1,3,4] | 3522.7 (3536.8) [1,2,4] | 4444.3 (4796.2) [1,2,3] | *<0.0001* |
| Folate | 157.3 (88.7) [2,3,4] | 190.6 (95.4) [1,3,4] | 229.6 (106.9) [1,2,4] | 308.3 (133.4) [1,2,3] | *<0.0001* |
| Vit B12 | 1.5 (1.5) [2,3,4] | 1.7 (1.6) [1,3,4] | 2.0 (1.8) [1,2,4] | 2.6 (2.1) [1,2,3] | *<0.0001* |

Q-quartile; median, SD- standard deviation; [1,2,3,4] indicate statistically significant results between quartiles (p-value ,0.05
